# Anaerobic Co-Culture of Mesenchymal Stem Cells and Anaerobic Pathogens - A New *In Vitro* Model System

**DOI:** 10.1371/journal.pone.0078226

**Published:** 2013-11-04

**Authors:** Katja Kriebel, Anne Biedermann, Bernd Kreikemeyer, Hermann Lang

**Affiliations:** 1 Katja Kriebel Department of Operative Dentistry and Periodontology, University Rostock, Rostock, Germany; 2 Anne Biedermann Department of Operative Dentistry and Periodontology, University Rostock, Rostock, Germany; 3 Bernd Kreikemeyer Institute of Med. Microbiology, Virology and Hygiene, University Rostock, Rostock, Germany; 4 Hermann Lang Department of Operative Dentistry and Periodontology, University Rostock, Rostock, Germany; National Institutes of Health, United States of America

## Abstract

**Background:**

Human mesenchymal stem cells (hMSCs) are multipotent by nature and are originally isolated from bone marrow. In light of a future application of hMSCs in the oral cavity, a body compartment with varying oxygen partial pressures and an omnipresence of different bacterial species i.e. periodontitis pathogens, we performed this study to gain information about the behavior of hMSC in an anaerobic system and the response in interaction with oral bacterial pathogens.

**Methodology/Principal Findings:**

We established a model system with oral pathogenic bacterial species and eukaryotic cells cultured in anaerobic conditions. The facultative anaerobe bacteria *Fusobacterium nucleatum*, *Porphyromonas gingivalis* and *Aggregatibacter actinomycetemcomitans* were studied. Their effects on hMSCs and primary as well as permanent gingival epithelial cells (Ca9-22, HGPEC) were comparatively analyzed. We show that hMSCs cope with anoxic conditions, since 40% vital cells remain after 72 h of anaerobic culture. The Ca9-22 and HGPEC cells are significantly more sensitive to lack of oxygen. All bacterial species reveal a comparatively low adherence to and internalization into hMSCs (0.2% and 0.01% of the initial inoculum, respectively). In comparison, the Ca9-22 and HGPEC cells present better targets for bacterial adherence and internalization. The production of the pro-inflammatory chemokine IL-8 is higher in both gingival epithelial cell lines compared to hMSCs and *Fusobacterium nucleatum* induce a time-dependent cytokine secretion in both cell lines. *Porphyromonas gingivalis* is less effective in stimulating secretion of IL-8 in the co-cultivation experiments.

**Conclusions/significance:**

HMSCs are suitable for use in anoxic regions of the oral cavity. The interaction with local pathogenic bacteria does not result in massive pro-inflammatory cytokine responses. The test system established in this study allowed further investigation of parameters prior to set up of oral hMSC *in vivo* studies.

## Introduction

Human mesenchymal stem cells (hMSCs) have a multipotent phenotype. They are defined by their capacity to attach to plastic culture surfaces, express specific surface markers and their differentiation program. This results in osteogenic, chondrogenic, and adipogenic lineages [1]. Adult hMSCs were originally isolated from bone marrow [2]. The presence of stem cells was illustrated in a number of adult tissues such as adipose, muscle, dermis, periosteum, gingiva, synovial membrane, synovial fluid and articular cartilage [3]–[5].

The concentration of oxygen can vary from 1–7% in bone marrow and 10–15% in adipogenic tissue [6], [7]. In many of those adult tissue environments hypoxic conditions could be an essential element of the hMSC life cycle. *In vitro* studies revealed increased expression of osteoblastic and adipogenic differentiation markers under hypoxic conditions [8], [9]. Another characteristic of hMSCs is the ability to repair and process the tissue by secreting a large number of angiogenic growth factors, anti-apoptotic factors and anti-inflammatory cytokines [10]. HMSCs showed immunosuppressive properties and have been suggested to inhibit different kinds of immune cells, including T cells, B cells, dendritic cells, and natural killer (NK) cells [11], [12]. In a mouse model Mei and collaborators demonstrated that hMSC improve the survival of sepsis by reducing inflammation (IL-10, IL-6) and enhancing the bacterial clearance [13]. These effects could result in a positive influence on the surrounding tissue during regeneration and hMSCs are a promising approach for a wide range of applications in regenerative medicine. Studies over the past several years described the host-pathogen interactions for various epithelial cells in intestinal, respiratory, and genitourinary tracts [14].

Infections of human body compartments caused by microbial pathogens can lead to a large spectrum of disease manifestations ranging from mild and self-limited to fulminant and lethal, or can be chronic and debilitating. An essential step in the process of infection caused by many pathogenic bacteria is the adherence and internalization of the bacteria on and in the host cells. The human oral cavity is constantly exposed to microorganisms interacting with hard and soft tissues [15]. The interaction between gingival epithelial cells (GECs) or gingival fibroblast (GF) and oral microorganism like *Fusobacterium nucleatum*, *Porphyromonas gingivalis* and *Aggregatibacter actinomycetemcomitans* is a well explored field [16]–[18]. These bacterial species were also shown, via their stimulating activity on oral epithelial cells, to act as potent inducers of pro-inflammatory cytokine release, including interleukin IL-1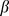, IL-6 and IL-8 [19], [20]. However, thinking about a future application of hMSCs in the human body, it is clear that not much is known about a potential hMSC-bacteria interaction.

*Fusobacterium nucleatum* is a gram negative obligatory anaerobe bacterium, which is described as commensal or opportunistic oral pathogen. It is capable of invading gingival epithelial cells and showed differences in internalization depending on cell type and bacteria subspecies [21]. The gram negativ bacterium *Porphyromonas gingivalis* is also able to adhere to and invade in gingival epithelial cells [22]. *Fusobacterium nucleatum* and *Porphyromonas gingivalis* survive and replicate in the cytoplasm of the immortalized epithelial cells and oral fibroblast resulting in a survival of the tissue [23]. A co-infection model uncovered that *Fusobacterium nucleatum* enhances the invasion of *Porphyromonas gingivalis* in gingival epithelial cells and human aortic endothelial cells [24]. Subsequent to bacterial infection, epithelial cells recruit phagocytic cells by producing chemotactic substances like interleukin-8 (IL-8). The secretion of IL-8 from epithelial cells is stimulated by the presence of *Fusobacterium nucleatum* and showed an increase over time [25]. Likewise *Porphyromonas gingivalis* triggered a reduced IL-6 and IL-8 transcription in human gingival fibroblasts [26].

The gram-negative facultative anaerobic bacterium *Aggregatibacter actinomycetemcomitans* is another important bacterial species associated with oral infections. Epithelial cell invasion of this species is mediated by microfilaments [27]. Beside *Fusobacterium nucleatum*, *Porphyromonas gingivalis* and *Aggregatibacter actinomycetemcomitans* were shown to increase the invasion of *Pseudomonas aeruginosa* into epithelial cells [28]. Thus, like the other species mentioned above, an interaction for species like *Aggregatibacter actinomycetemcomitans* with hMSCs in the oral cavity is very likely.

Stem cells act as a repair system and, under pathological conditions, such as tissue injury, these cells are mobilized towards the site of damage and thus get in contact with bacteria. The interaction of oral pathogens with epithelial cells is described well [22], [29]–[31], but the bacterial influence on stem cells and the question how the hMSC and bacteria can interact is only poorly understood.

Our aim was to investigate the behavior of bacteria and eukaryotic cells under these conditions and to gain insight into hMSC response towards presence of oral pathogenic bacteria.

## Results

### Cultivation of hMSC and GECs under Anaerobic Conditions

**Figure 1:**
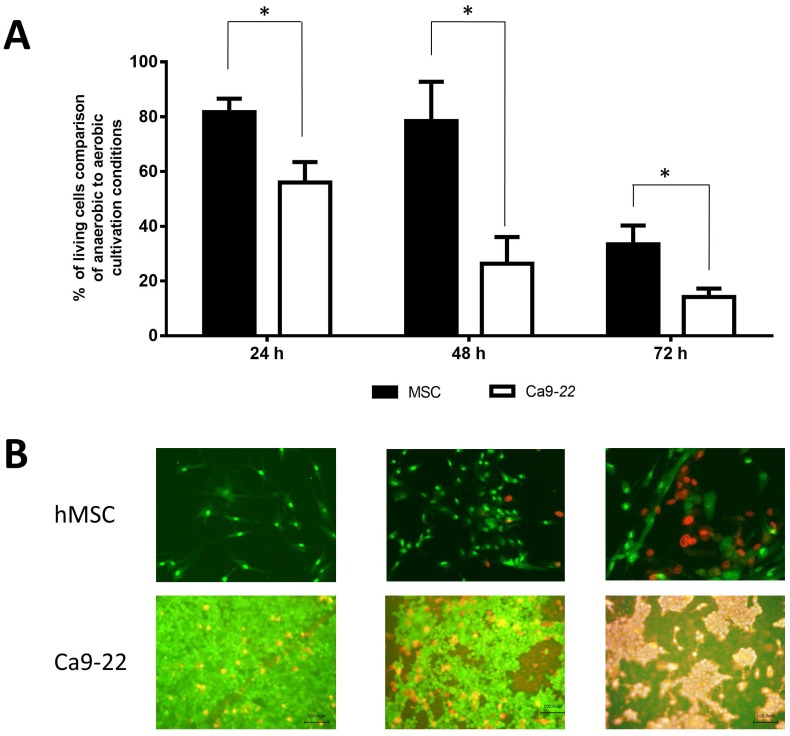
Survival of hMSC and Ca9-22 under aerobic and anaerobic conditions. (A) Percentage of living cells in comparison of aerobic to anaerobic cultivation conditions. The significance was calculated via Mann-Whitney-U test. P≤0.05 was considered as significant. (B) Immunofluorescence microscope images of hMSC and Ca9-22 stained with Live/Dead dyes after 24 h, 48 h and 72 h after anaerobic conditions.

In a first series of experiments we analyzed the viability of cells during growth under anaerobic conditions. We compared the viable cell numbers recovered after incubation in aerobic and anaerobic atmosphere. Figure 1A shows that hMSC were able to survive to a significant extent over three days under anaerobic conditions. After 48 h of anaerobic growth 80% of the cells were still viable. In comparison the gingival epithelial cell lines Ca9-22 as well as HGEPC did not tolerate anoxic conditions (Figure 1A and Figure S1 ). A significant decrease of viability is apparent after two days. After three days of subsequent anaerobic incubation almost 40% of the hMSCs were viable, whereas only 14% to 20% of the gingival epithelial cells survived this treatment (Figure 1A and Figure S1).

To confirm these results and visually document the cellular behavior, cells were stained with Live/Dead dye and monitored via fluorescence microscopy (Figure 1B).

### Growth of Bacteria in the Cell Culture Medium

**Figure 2:**
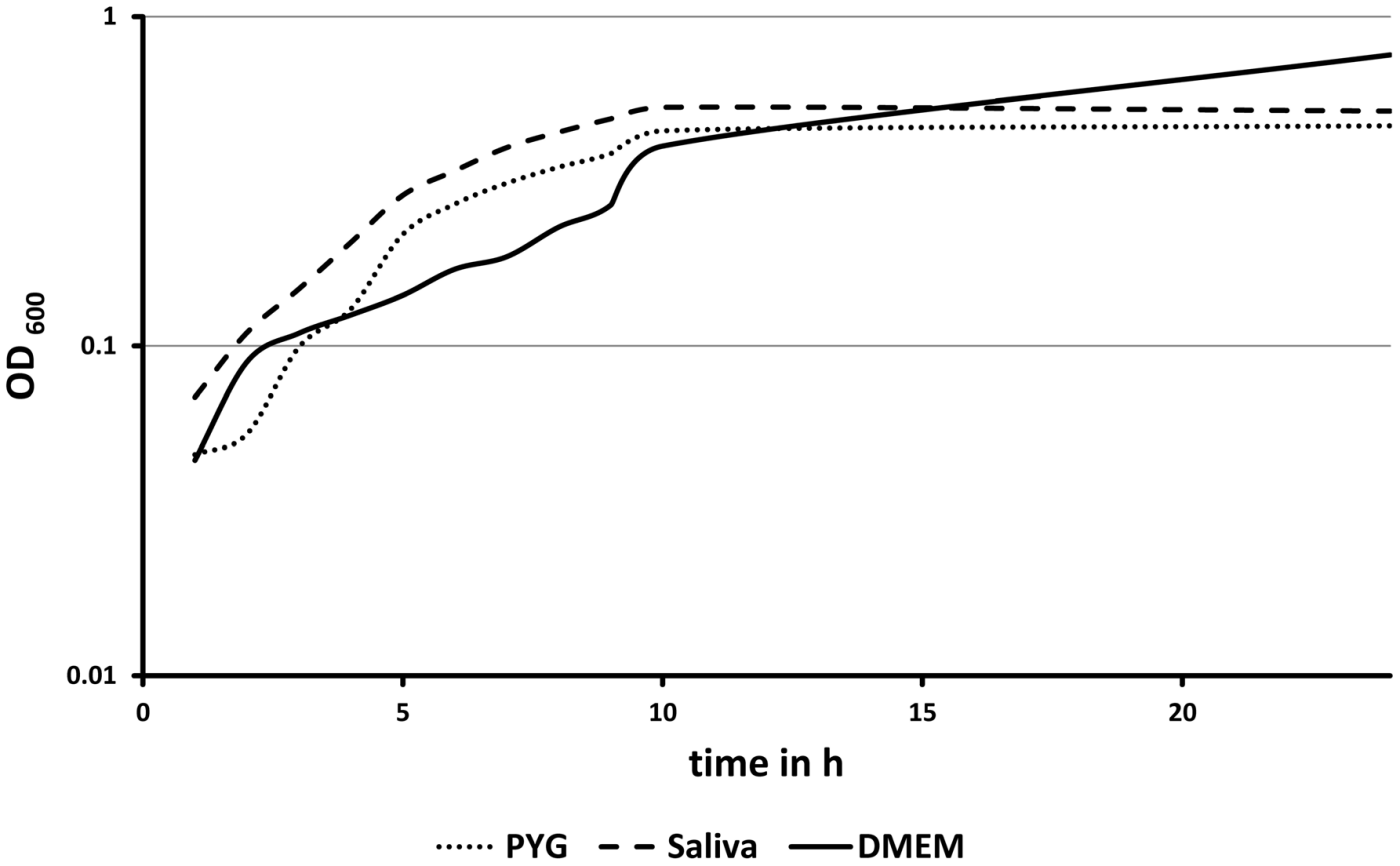
Growth curve of *Porphyromonas gingivalis*. The microorganism were tested for growth in bacteria optimized PYG medium (dotted line), in artiffcial saliva (broken line), which simulated an *in vivo* situation, and in the cell culture medium DMEM (solid line).

Next we studied the growth behavior of the oral pathogenic bacterial species *Porphyromonas gingivalis*, *Fusobacterium nucleatum*, and *Aggregatibacter actinomycetemcomitans* in cell culture medium. This was a prerequisite prior to any bacterial-cell co-culture experiments. We compared growth in (I) PYG medium, which is well adapted and is known to support growth of the species investigated, (II) artificial saliva, in order to simulate the *in vivo* situation, and (III) the cell culture medium DMEM, which so far was never investigated in its propensity to support bacterial growth. Figure 2 depicts one representative growth curve from three independent biological replicates for *Porphyromonas gingivalis* in the three media. It is apparent, that all media substantially support the growth of this species and no attenuation of calculated growth could be detected in DMEM. Almost the same final optical densities were reached.

Overall the growth rates (Table 1) in saliva and DMEM showed no significant difference. The growth behavior of *Fusobacterium nucleatum* and *Aggregatibacter actinomycetemcomitans* revealed no differences compared to *Porphyromonas gingivalis* (data not shown). The growth rates for these species are shown in Table 1.

**Table 1:** Growth rate in PYG, DMEM and artificial saliva over a time period of 24 h.

	DMEM	Saliva	PYG
*Fusobacterium nucleatum ATCC 23726*	0.20±0.07	0.14±0.05	0.27±0.05
*Fusobacterium nucleatum ATCC 25586*	0.16±0.09	0.16±0.01	0.21±0.12
*Porphyromonas gingivalis W50*	0.19±0.02	0.12±0.03	0.21±0.11
*Porphyromonas gingivalis W83*	0.20±0.04	0.13±0.03	0.34±0.03
*Aggregatibacter actinomycetemcomitans*	0.15±0.06	0.22±0.02	0.31±0.03
*Aggregatibacter actinomycetemcomitans HK*	0.13±0.03	0.19±0.02	0.36±0.03

Growth rate in PYG, DMEM and artificial saliva over a time period of 24 h.

### The Interaction between Bacteria and Cells

**Figure 3:**
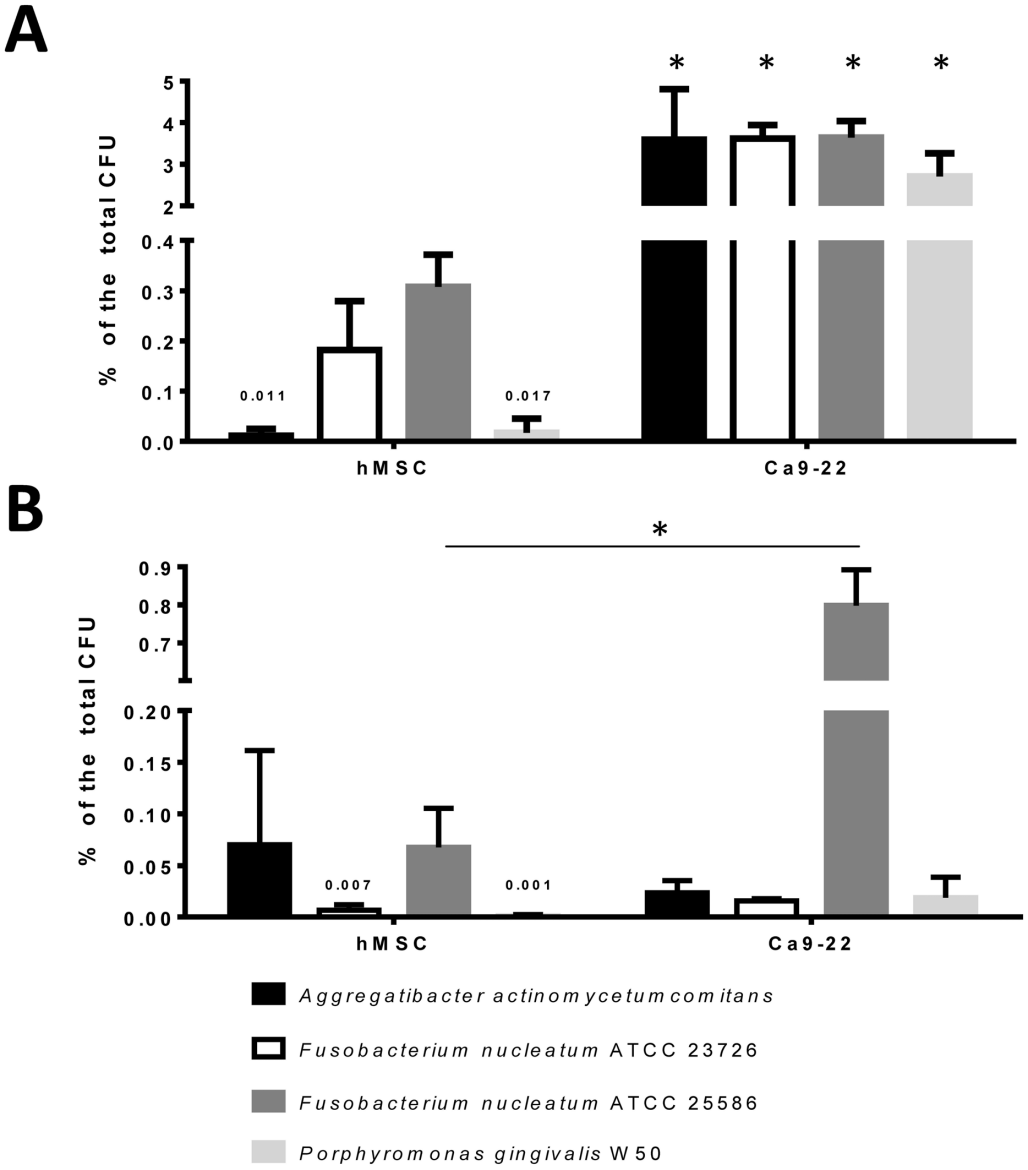
Quantification of the interaction of cells and oral bacteria. (A) Adherence and (B) Internalization of *Aggregatibacter actinomycetemcomitans*, *Porphyromonas gingivalis*, *Fusobacterium nucleatum* ATCC 23725 and *Fusobacterium nucleatum* ATCC 25580 in co-culture with human mesenchymal stem cells hMSC (black bars) and gingival epithelial cell line Ca9-22 (white bars) under anoxic conditions.

Since all bacterial species were metabolically active and viable in DMEM under anaerobe conditions we next set up the co-culture experiments with the gingival epithelial cells and the hMSCs, again using anaerobic conditions. We determined the extent of bacterial adherence to and internalization into the eukaryotic cells, using a MOI of 1∶100. Figure 3A illustrates that for all bacterial species under investigation roughly 3% of the inoculum was able to firmly adhere to the gingival epithelial cells Ca9-22. In contrast hMSCs did not support a substantial cellular adherence of the bacteria, as only below 0.5% of the inoculum was able to attach. Consequently, only negligible numbers of bacteria were found internalized in the hMSCs (Figure 3B). Internalization rates of the bacteria into the Ca9-22 cells were as low as measured for the hMSCs, except for *Fusobacterium nucleatum* ATCC 25586, which invaded the epithelial cells in significant numbers. The human gingival primary epithelial cells (HGPEC) were phenotypically almost identical in their response to *Fusobacterium nucleatum* and *Porphyromonas gingivalis* challenge if compared to Ca9-22 cells (Figure S2).

**Figure 4:**
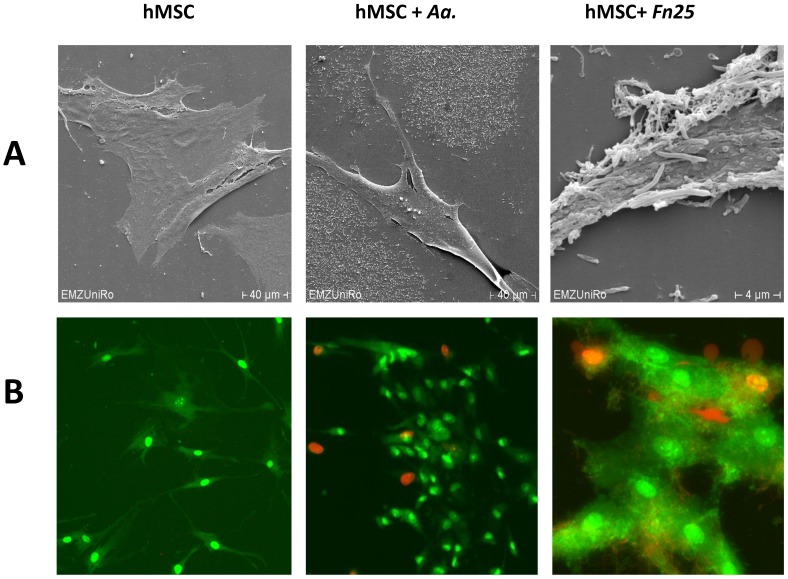
Direct interaction of oral bacteria on hMSC surface. (A) Scanning electron microscope and (B) immunofluorescence microscope pictures. The immunofluorescence microscope samples were stained with Live/Dead dyes. Live cells are stained in green, dead cells light up in red.

As a second technique to visualize the interaction of the bacteria with the eukaryotic cells we used SEM and fluorescence microscopy. Figure 4A depicts a direct interaction of the bacteria with the hMSCs cell surface. The immunofluorescence pictures (Figure 4B) after live/dead staining support the notion that both, cells and bacteria stay viable in the co-culture conditions for up to 24 hours.

Together these data underscore that hMSCs are not significantly targeted by the pathogenic bacteria. However, to support the hypothesis further experiments are needed. Primary and permanent gingival epithelial cells much better support bacterial adherence, but strain-specific differences exist for the internalization capacity of the *Fusobacterium nucleatum* strains.

### Interleukin Secretion

After investigating the interaction of the bacteria with hMSCs, Ca9-22, and HGPEC cells we determined the time-dependent induction of cytokine secretion under anaerobic co-culture conditions. To exclude an influence of cellular or bacterial growth and viability on interleukin secretion, 24 h co-incubation was used as the latest time point. The values of non-infected cells were measured and subtracted. The cell and bacterial viability monitoring proved a stable MOI throughout these experiments. Thus, decreased and differential cellular viability in anaerobic atmosphere did not effect the results in these experimental setting.

**Figure 5:**
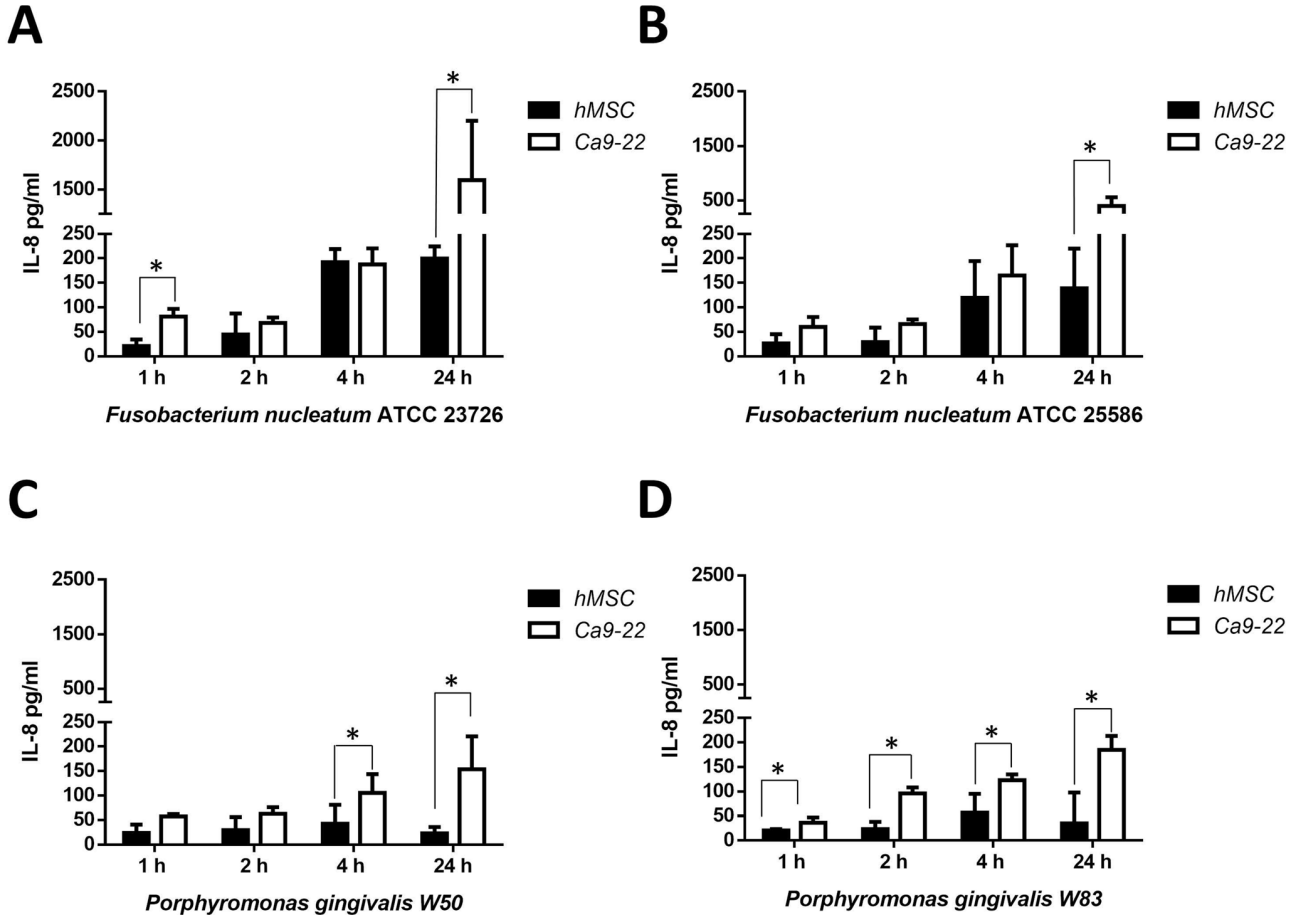
Enzyme-linked immunosorbent assay of IL-8. The hMSCs and Ca9-22 cells were challenged with a MOI of 1∶100 for 1, 2, 4 and 24 h with the microorganism (A) *Fusobacterium nucleatum* ATTCC 23726 (B)*Fusobacterium nucleatum* ATTCC 25586 (C) *Porphyromonas gingivalis* W50 or (D) *Porphyromonas gingivalis* W83. The Enzyme-linked immunosorbent assay was performed with supernatant from MSC (black bars) and Ca9-22 (white bars) after the indicated time points.

Commercially available ELISA assays were used to quantify IL-8 and IL-10 secretion during infection. The secretion of the PMN chemotactic substance IL-8 was overall stronger in all cell types in the presence of *Fusobacterium nucleatum* strains if compared to *Porphyromonas gingivalis* infected hMSCs, Ca2-99, and HGPEC cells (Figure 5, Fig. S3A ). hAdditionally, a time-dependent increase of IL-8 amounts was shown for all investigated strains during infection with gingival epithelial cells and primary epithelial cells. The IL-8 secretion was stimulated in hMSCs infected with *Fusobacterium nucleatum* over time, whereas *Porphyromonas gingivalis* infection of hMSCs did not induce a time-dependent IL-8 secretion. The *Porphyromonas gingivalis* induced IL-8 secretion was significantly higher in gingival epithelial cells compared to hMSCs.

**Figure 6:**
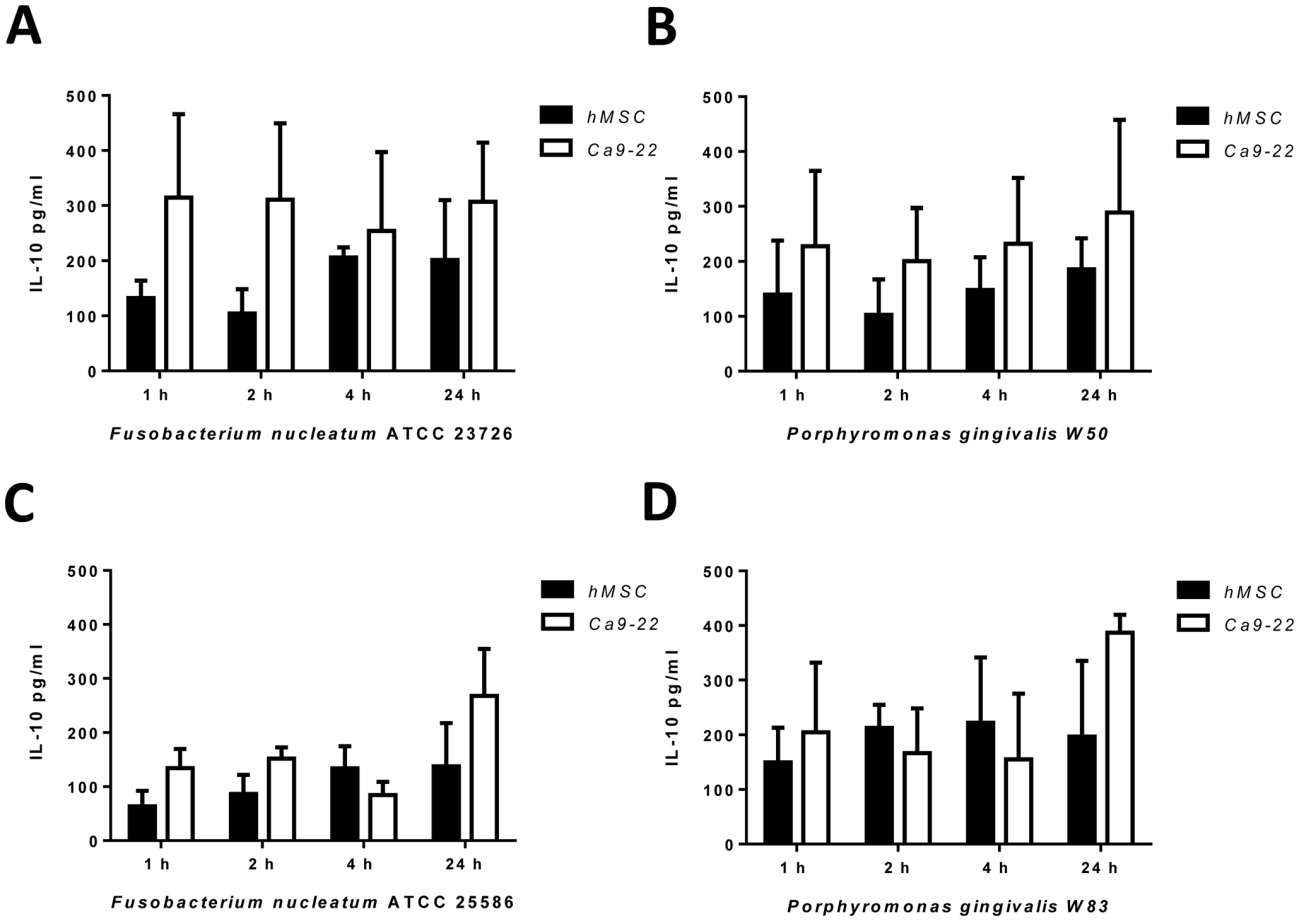
Enzyme-linked immunosorbent assay of IL-10. The cells were challenged with a MOI of 1∶100 for 1, 2, 4 and 24 h with the microorganism (A) *Fusobacterium nucleatum* ATTCC 23726 (B)*Fusobacterium nucleatum* ATTCC 25586 (C) *Porphyromonas gingivalis* W50 and (D) *Porphyromonas gingivalis* W83. The Enzyme-linked immunosorbent assay was performed with supernatant from MSC (black bars) and Ca9-22 (white bars) after the indicated time points.

Upon bacterial host cell contact, all bacterial species led to a IL-10 secretion, which is shown in Figure 6 and Figure S3B. Despite apparent differences, no statistical difference was reached, suggesting a rather uniform IL-10 secretion after bacterial infection.

## Discussion

In this study we established and validated an anaerobic *in vitro* co-culture system of periodontal pathogens and mesenchymal stem cells. In previously described studies of anaerobic microorganism and eukaryotic cells the incubation of the co-culture was performed under aerobic conditions and anaerobic microorganism had to tolerate the oxidative stress condition. To the best of our knowledge no model system in an environment without oxygen exists. The aerotolerance of strictly anaerobic microorganisms like *Porphyromonas gingivalis* is very low. After 2 h (37°C, 5% CO_2_) only 50% of the inoculum remaind viable [32]. Furthermore, primary isolates of rat marrow derived mesenchymal stem cells (rMSCs) maintained a greater number of colonies and proliferated more rapidly in an oxygen reduced atmosphere [6]. Other *in vitro* studies revealed that hMSCs had an approximately 30-fold higher expansion over 6 weeks without loss of multi-lineage differentiation capabilities in such hypoxic atmosphere [9]. The hypoxic preconditioning of hMSC reduced cell death and apoptosis of implanted cells [33].

Therefore it was promising to investigate the survival of hMSCs and primary as well as permantent gingival epithelial cells in an anaerobic atmosphere. The results proved that hMSC had an increased potential to adapt to the anaerobic conditions compared to differentiated cells (i.e. gingival epithelial cells Ca9-22 and human gingival primary epithelial cells HGPEC ). Also alveolar cells grown under hypoxic conditions showed a disruption of cytoskeleton integrity and apoptosis [34]. This indicated the possibility, that hMSC *in vivo* get in contact and adapt to hypoxic or anoxic conditions.

The adaption of eukaryotic cells to the bacterial growth medium was not possible. Hence we tested the ability of bacteria to grow in medium optimized for cell culture. The microorganism showed a reduction of the optical density after 24 h, a typical growth curve and stayed viable over 24 h, but the capacity of the biofilm formation was reduced (data not shown). To compensate the suboptimal conditions for the microorganism the co-cultivation was analyzed in the anaerobic atmosphere. While the anaerobe *Fusobacterium nucleatum* showed the capacity of adaption to a reduced oxygenated environment [35], *Porphyromonas gingivalis* is only able to survive and growth under aerated conditions in the presents of *Fusobacterium nucleatum* [36]. We showed that the co-culture of hMSCs and oral pathogenic bacteria in an anaerobic atmosphere is possible over 24 h hours in cell culture medium.

The validated model was used to perform the adherence and invasion assays of the anaerobe microorganism in the hMSCs and gingival epithelial cells without a major loss in viability. Previous studies on adherence and invasion of oral pathogens into various cell types like gingival fibroblasts or heart endothelial cells are well described [31], [37]–[40], but the interaction between hMSC and anaerobe bacteria is still poorly understood. The characterization of the adherence and internalization for *Porphyromonas gingivalis*, *Fusobacterium nucleatum* and *Aggregatibacter actinomycetemcomitans* with hMSCs demonstrated, that the adherence as well as the invasion of bacteria was significantly less pronounced in co-cultures with stem cells (hMSCs) compared to differentiated cells (gingival epthelial cells). In the oral cavity the mechanisms of adherence and internalization are essential for bacterial pathogenesis. The ability of invasive bacterial species to manipulate the host cell receptors and recruit a number of effector molecules is important to survive the host immune response [41].

To determine the cellular response of the hMSCs and gingival epithelial cells we analyzed the secretion of IL-8, which is presented in the inflammatory milieu, and IL-10 an anti-inflammatory cytokine. The inflammatory response of gingival fibroblasts, the connective-tissue cells located in the apical gingiva, and periodontal ligament fibroblasts, located in the periodontal ligament, was investigated and showed different effects of viable *Porphyromonas gingivalis* on the expression of genes associated with inflammation [42]. Viable *Porphyromonas gingivalis* triggers a higher secretion of IL-8 over 4 h and 24 h in the gingival epithelial cells compared to heat-killed components in the study from Stathopoulou *et al*. [20]. In our study the viable oral pathogens showed a reduced IL-8 secretion in hMSCs compared to the gingival epithelial cells and human gingival primary epithelial cells using the anaerobe co-cultivation system. For *Porphyromonas gingivalis* the results could be based on their ability to degrade cytokines via protease activity post secretion [43]. The time dependent increase of IL-8 secretion of gingival epithelial cell in co-culture with *Fusobacterium nucleatum* was shown in aerobic conditions and we observed the same effects in the anaerobic atmosphere [20]. The low IL-8 secretion of the hMSCs could be explained by the immunomodulatory effects of hMSC [44]. Secretion of interleukin 10 was found for hMSC during cell cell contact with T-lymphocyte and we showed the induction by living bacteria [45]. During the incubation the hMSC illustrate a similar IL-10 secretion like the gingival epithelial cells.

It is noteworthy to mention that no significant difference in the phenotypes of the permanent gingival epithelial cell line Ca9-22 and its primary counterpart HGPEC could be determined throughout our experiments. This suggest that all currently published date are comparable and supports the motion that permanent gingival epithelial cells lines are suitable models for basic research.

Summarizing our results, we established an anaerobic culture system with anaerobic pathogenic bacteria and cell lines in various states of differentiation. The compared cell lines showed variable reactions to the exposure of bacteria i.e. adherence, internalization and interleukin secretion in the anaerobic test system. Apparently hMSCs are more tolerant towards bacterial infection and underline the application as promising tool in regenerative medicine in human body compartments exposed to bacteria.

## Materials and Methods

### Cells and Culture Conditions

The Human Mesenchymal Stem Cells were derived of bone marrow from donors of the Department of Cardiac Surgery of the University of Rostock. The hMSC isolation was performed as previously described [46]. The participants gave their written consent to participate in this study and this consent procedure was approved by the ethics committee of the University of Rostock, Germany (A 2011 119). The cells were cultured in Dulbecco modified Eagle medium (DMEM, Invitogen, Karlsruhe, Germany) with 10% heat-inactivated fetal bovine serum (FBS, PAA Laboratories Cölbe) and grown at 37°C, in 5% CO_2_. Additionally the medium was changed every four days. Before the experiments were started, the adherent cells were dissociated with Accutase (PAA Laboratories, Cölbe), counted and 4*10^3^ 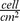 attached in 24 or 96 well plates (Greiner Bio-one, Frickenhausen). The human gingival epithelial cell line Ca9-22 [47] was provided by the German Cancer Research center Heidelberg. These cells were grown in DMEM (Invitogen, Karlsruhe, Germany) supplemented with 10% heat-inactivated fetal bovine serum in 75 cm^2^ cell culture flask (Greiner Bio-one, Frickenhausen) in a CO_2_ incubator at 37°C. For experiments, cells were grown to subconfluence in this medium and seeded in 24 or 96 well plates (Greiner Bio-one, Frickenhausen). The human gingival primary epithelial cells (HGPEC) were provided by the CELLnTEC Advanced Cell Systems AG from Switzerland. The cells were isolated from the human adult gingiva and pooled from 3 or more donors. The cultivation was done in commercial available CnT-24 medium with the adequate supplements at 37°C, in 5% CO_2_. For experiments, cells were grown to subconfluence in CnT-24 medium and seeded in 24 or 96 well plates (Greiner Bio-one, Frickenhausen). The seeding density was 4*10^3^ 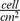 in DMEM supplemented with 10% FBS.

### Bacterial Strains and Culture Conditions

The strain *Aggregatibacter actinomycetemcomitans* DSMZ 11123 (Aa) was cultured in brain heart infusion medium (BHI, Invitrogen) at 37°C under a 5% CO_2_-20% O_2_ atmosphere. The strains *Porphyromonas gingivalis* W50, *Porphyromonas gingivalis* W83, *Fusobacterium nucleatum* ATTCC 23726 and *Fusobacterium nucleatum* ATTCC 25586 were grown in BHI supplemented with 5 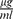 hemin and 1% vitamin K in an anaerobic atmosphere (10% CO_2_, 10% H_2_, 80% N_2_). The bacteria were grown overnight to mid-logarithmic phase, centrifuged, washed with PBS and resuspended in DMEM medium to a cell number of 10^7^ cells per ml. (A multiplicity of infection (MOI) of one cell to 100 bacteria was used.) All strains were purchased from commercial providers (DMSZ, Braunschweig, Germany and ATCC, Manassas, USA).

### Co-culture of Bacteria and Cells

The direct interaction between bacteria and cells was investigated in 24 well plates. Overnight cultures of the bacteria were centrifuged and adjusted to the MOI 1∶100 cells to bacteria in DMEM medium. The anaerobic atmosphere with 10% CO_2_, 10% H_2_ and 80% N_2_ at 37°C was used as culture condition.

### Cellular Viability Assay

Cellular viability was determined by Trypan Blue dye enumeration. The cells were seeded in a 24 well plates and exposed to the anaerobic conditions for 24 h up to 72 h, then trypsinized and resuspended in DMEM with 10% heat-inactivated FBS. After staining with Trypan Blue, viable cells were counted using a Neubauer counting chamber and a inverted light microscop.

### Growth Experiments

The overnight culture of the bacteria was centrifuged and adjusted to an optical density of 0.1 at 600 nm, which is equal to the colony forming unit of 10^7^ bacteria. In BHI Saliva and DMEM the bacteria were grown over a time period of 24 h.

### Documentation of Infection by Fluorescence and Scanning Electron Microscopy

The cell lines were seeded in 24-well polystyrene cell culture plates (Greiner Bio-One, Frickenhausen, Germany), each well containing a sterile, uncoated 13 mm diameter plastic microscope cover slip (Nunc, Wiesbaden, Germany) or sterile glass cover slip. After one to three days of incubation under anaerobic conditions the cells were washed with PBS. For fluorescence microscopy (BX60 microscope, Olympus, Hamburg, Germany) the samples were stained with Live/Dead dye (Molecular Probes, Eugene, Oregon). The interaction was documented with an attached digital camera (Leica, Solms, Germany).

For scanning electron microscopy (SEM) samples of bacteria with and without the cover slips samples were fixed after the indicated days of incubation for 24 h in a 2.5% glutardialdehyde solution. Afterwards the coverslips were washed with 0.1 M sodium acetate buffer (pH 7.3) and dehydrated in a graded series of ethanol. Subsequently, coverslips were subjected to critical point drying with CO_2_, sputter-coated with gold (thickness approx. 10 nm), and examined with a Zeiss DSM 960A electron microscope.

### Adherence and Internalization Procedures

For the experiment 4*10^3^ 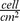 hMSC were cultured in DMEM and grown over two days to form a monolayer. The bacteria were grown in a preculture for 48 h in BHI supplemented with 5 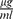 hemin and 1% vitamin K medium at 37°C under anaerobic atmosphere (10% CO_2_, 10% H_2_, 80% N_2_). The bacteria were concentrated by centrifugation and the pellet resuspended in PBS. The bacterial density was adjusted to 10^5^ cells per ml in DMEM medium without fetal calf serum (FCS) and added to the hMSC monolayer. After 2 h, cells were washed with PBS and subsequently detached by adding 200 µl 0.25% trypsin/0.5 mM EDTA for 10 min at 37°C. To quantify bound bacteria, cells were lysed with sterile distilled water and the number of bacteria in the lysate was assessed by viable counts. For testing the internalization of bacteria into the cells penicillin 100 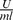 and streptomycin 0,1 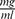 was added after 2 h and incubated for another 2 h. After 4 h the procedure established for adherence testing was used to count the internalized bacteria.

### Enzyme-Linked Immunosorbent Assay

The interleukin concentration of IL-8 and IL-10 in the supernatant was measured by Elisa using the commercially available kit BD OptEIA from BD Bioscience. The samples of the supernatant from the co-culture of after 1 h, 2 h, 4 h and 24 h. Samples were centrifuged and the supernatant was stored at −20°C. The analysis of the samples was done according to the manufacture instructions. The values of non-infected cells matching the investigation time points were measured and subtracted for each experiment. The supernatants were take from a minimum of three individual experiments.

### Statistical Analysis

Each experiment was performed in triplicate independent occasions and data were calculated in terms of mean +/− standard deviation. The p-Values were determined by the Mann-Whitney U Test and P-values less than 0.05 were considered as significant. An exception were the results of the human primary epithelial cells (HGPEC). These results shown in Figure S 1–3 were from two biological replicates with two technical replicates and no statistical analysis of the data was performed.

## Supporting Information

Figure S1 **Survival of HGPEC under aerobic and anaerobic conditions.** The survival of HGPEC is shown as percentage of living cells in comparison of aerobic to anaerobic cultivation conditions. The results of the HGPEC, presented here from two biological replicates with two technical replicates, each.
(TIF)

Figure S2 **Quantification of the interaction of HGPEC cells and oral bacteria.** Adherence and Internalization of *Porphyromonas gingivalis* W50 (white bars) and *Fusobacterium nucleatum* ATCC 25580 (black bars) in co-culture HGPEC under anoxic conditions was investigated. The results represent two biological replicates with two technical replicates.
(TIF)

Figure S3 **Enzyme-linked immunosorbent assay of IL-8 and IL-10.** (A) IL-8 concentration and (B) IL-10 concentration after incubation with the microorganism *Fusobacterium nucleatum* ATTCC 25586 (black bars)and *Porphyromonas gingivalis* W50 (white bars). The cells were challenged with a MOI of 1∶100 for 1, 2, 4 and 24 h with the microorganism. The Enzyme-linked immunosorbent assay was performed with supernatant after indicated time points. The results depicted are from two biological replicates with two technical replicates.
(TIF)
